# Breast cancer risk in papilloma patients: Osteopontin splice variants indicate prognosis

**DOI:** 10.1186/s13058-022-01561-9

**Published:** 2022-09-29

**Authors:** Piotr Ziółkowski, Marta Woźniak, Ahmad Mansour, Yu An, Georg F. Weber

**Affiliations:** 1 Department of Pathology, Wroclaw Medical University, Wroclaw, Poland; 2 Department of Pathology, University of Cincinnati, Cincinnati, OH USA; 3 Division of Biostatistics and Bioinformatics, Department of Environmental and Public Health Sciences, Cincinnati, OH USA; 4 College of Pharmacy, University of Cincinnati, 3225 Eden Avenue, Cincinnati, OH 45267-0004 USA

**Keywords:** Breast papilloma, Tumor progression marker, Immunohistochemistry, Breast cancer, Osteopontin

## Abstract

**Background:**

Papillomas of the breast pose challenges for treatment decisions as their risk for transformation to breast cancer is low but not negligible. To spare low-risk patients the burden of substantial treatment side effects, prognostic indicators are needed for cancerous progression. The secreted metastasis mediator Osteopontin (OPN) is a marker for breast cancer aggressiveness, and its variants are prognosticators for transformation in diverse premalignant breast lesions. Here, we test whether the presence of OPN-c or OPN-exon-4 in papillomatous lesions may reflect progression risk.

**Methods:**

By immunohistochemistry, we analyze OPN-c and OPN-exon-4 in papillomas from 114 women as well as correlations between staining and progression. In departure from prior spliced OPN biomarker publications, we utilize novel monoclonal antibodies.

**Results:**

Fewer than 5% of OPN-c pathology score 0–1 (intensity) versus almost 18% of score 2–3 experienced cancer in follow-up. Nine of 12 women, who progressed, had pathology scores of 2–3 for OPN-c intensity at the time of initial diagnosis, and none had a score of 0. When developing a combined risk score from intensity plus percent positivity for OPN-c, the progression risk for patients with low score was 3.2%, for intermediate score was 5.7%, and for high score was 18.8%. Papillomas in patients, who were later diagnosed with cancer in the contralateral breast, displayed stronger staining positivity than non-progressors.

**Conclusion:**

OPN splice variant immunohistochemistry on biopsies of breast papillomas will allow counseling of the patients on their risk to develop breast cancer at a later time.

**Supplementary Information:**

The online version contains supplementary material available at 10.1186/s13058-022-01561-9.

## Introduction

Among benign breast biopsies, papillomas account for about 5% of the cases [1, 2]. Morphologically, these papillary lesions of the breast are characterized by the presence of arborizing fibrovascular cores derived from the wall of the ducts, which are often distended by the lesions. The fibrovascular cores are, in turn, lined by layers of epithelial cells, with or without a complete layer of intervening myoepithelial cells [3]. Although criteria to distinguish papillomas from papillary carcinomas have long been described in the literature [4], on core needle biopsy, a differentiation between papilloma and papillary ductal carcinoma in situ may be difficult [2].

Fragments of a benign papilloma in a breast biopsy are considered a lesion of uncertain malignant potential (B3 in the European classification), and excision is mostly recommended [5], although its necessity has been subject to debate [6–8]. A single papilloma imparts a cancer risk similar to conventional proliferative fibrocystic change. It has been approximated at 2–6% above papilloma-free women [1, 9]. Multiple papillomas constitute a proliferative breast disease subset having unique clinical and biologic behavior [1] and encompassing an increased likelihood for transformation [10]. A major concern in the management of breast papilloma is the carcinoma upgrade rate consecutive to biopsy, which various sources have estimated in the range of 7–25% [11, 12], as well as the associated predictive factors [13, 14]. A molecular diagnostic that informs the patient whether she is at high or low risk for developing breast cancer from a current papilloma can substantially facilitate the decision on follow-up treatment.

The cytokine Osteopontin (OPN, SPP1) constitutes the most abundantly secreted phosphoprotein in breast cancer, which also supports invasive behavior. The gene product is subject to alternative splicing selectively in cancer, which deletes exon 4 (to generate Osteopontin-c) from the unspliced form (called Osteopontin-a); Osteopontin-b (lacking exon 5) is absent from healthy and transformed breast tissue. In a prior meta-analysis, we reported that pan-Osteopontin (no discrimination among splice forms) is correlated with premalignant progression in breast and other transformations [15, 16]. In a histopathology investigation of various premalignant lesions, we have more recently shown that a combination of Osteopontin-c and Osteopontin exon 4 can serve as an effective predictor for transformation risk and later death [17]. Here, we focus selectively on papillomatous growths, and we utilize monoclonal antibodies for the immunohistochemistry.

## Materials and methods

### Patients

This study investigated biopsies from a total of 114 women with premalignant breast lesions and follow-up information over 5–11 years, comprising 49 patients from Wroclaw, Poland, and 65 patients from Cincinnati, USA. In addition, 7 tissues from Wroclaw were stained but either had no follow-up information or encountered death from other causes and were therefore excluded from evaluation. These cases comprise all papillomas with the necessary follow-up information that were accessible to the authors from both study sites. To assure uniformity of the analysis, the tissues were stained and read by two pathologists in the same institute, and the initial diagnoses were confirmed by them. All the evaluations were made based on international standards and classifications (specifically, College of American Pathologists [CAP] for USA and the standards of the Polish Society for Pathologists, which were also based on CAP outlines). The research project was approved by the ethics committees at Wroclaw Medical University, Poland, and at the University of Cincinnati, USA.

### Immunohistochemistry

**Fig. 1:**
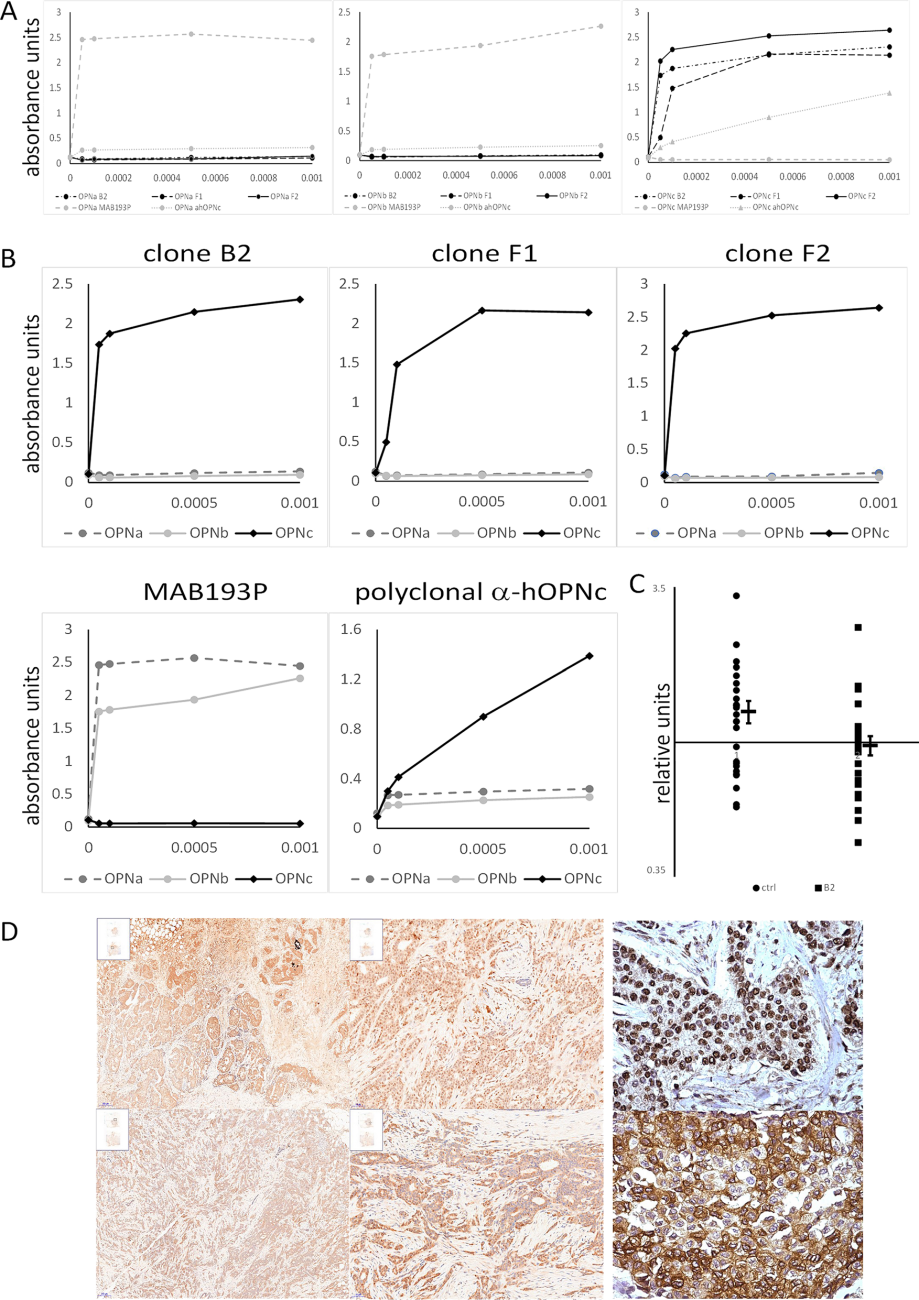
Anti-OPNc monoclonal antibody validation. **A** Solid-phase ELISA for antibody-binding to GST-Osteopontin splice variants. B2, F1, and F2 are individual clones of the rabbit monoclonal antibodies to OPN-c. The antibody ahOPNc is the polyclonal chicken antibody to OPN splice variant-c. The antibody MAB193P recognizes exon 4. The y-axis indicates absorbance. The antibodies were tested for binding to OPN-a (left panel), OPN-b (middle panel), or OPN-c (right panel). **B** The antibodies utilized in **A**) were tested in further solid-phase ELISA binding to the distinct splice variants of GST-Osteopontin. **C** Antibody neutralization of soft agar colony formation. Stably transfected MCF-7 OPN-c cells were plated in soft agar under standard conditions [31]. On day 0, 2 µg of antibody (clone B2) was added per plate and 0.6 µg every other day with medium (the control received only medium). The clone sizes were measured on day 11 as relative units [32]. The y-axis is displayed on logarithmic scale. The error bars are mean ± sem. The difference is significant according to the *t* test (*p* = 0.004). The other antibodies gave similar results (not shown). **D** Antibody validation in immunohistochemistry of breast cancers. The upper panel of slides is stained with anti-OPN-exon-4 antibody MAB193P (5× and 20× magnification, followed by a zoomed-in 20×), while the lower panel slides are probed with anti-OPN-c antibody clone F2 (5× and 20× magnification, followed by a zoomed-in 20×)

Previous immunohistochemistry studies of Osteopontin-c, reported by us and others [17–23], were conducted with the polyclonal chicken IgY that had been generated by us and made available to other researchers through Gallus Immunotech, later Exalpha Biologicals. The polyclonal rabbit antibody LF161 (obtained from Larry Fisher, NIH) served as a marker for Osteopontin exon 4. Here, we use monoclonal antibodies. Exon 4 is recognized by the mouse monoclonal antibody MAB193P (obtained from BBI Solutions). Using the immunizing peptide ac-SEEKQ|NAVS (the vertical line marks the Osteopontin-c splice junction) coupled to KLH via a C-terminal cysteine, we generated 3 clones of rabbit monoclonal anti-Osteopontin-c. All of the monoclonal antibodies displayed strong and selective reactivity with their target antigen, and they showed neutralizing activity in soft agar colony formation. Dissimilar to the polyclonal antibodies, the monoclonal OPN-c stain located predominantly to the cytoplasm, while the exon 4 stain was nuclear and cytoplasmic (Fig. 1).

For each antibody, formalin-fixed and paraffin-embedded biopsy specimens from papillomatous lesions were cut on a microtome. The antibodies used in this study, after blocking endogenous peroxidase for 10 min with DAKO Peroxidase Blocking Solution and non-specific proteins for 15 min with DAKO Protein-Block Serum-Free, were monoclonal anti-OPN-c rabbit Ig clone F2 (Georg F. Weber recognizes the Osteopontin-c splice junction), and murine monoclonal anti-OPN-exon-4 Ig MAB193P (BBI Solutions recognizes OPN-a and OPN-b, but in the breast is indicative of OPN-a because OPN-b is absent). The primary antibodies were applied at 1.25 μg/ml (F2) or 2.2 μ/ml (MAB193P) dilution for 30 min followed by DAKO EnVision + System HRP-labeled polymer for 30 min. Color development was achieved with DAB + chromogen from DAKO for 5 min and consecutive counterstaining with hematoxylin for 1 min. For each antibody, the tissues were scored according to staining intensity (0, 1, 2, or 3) and percent positivity (0, 1, 2, or 3). In addition to analyzing the indicators in their original scale, we dichotomized the immunohistochemical biomarkers into low (0–1) or high (2–3). We have previously found this dichotomizing to strengthen the power of the analysis. Further, it enables the development of a clear-cut risk score [17, 20]. In those predecessor studies, a cutoff between 0 and 1 was found to place many low-risk patients into the high group, whereas a cutoff between 2 and 3 would have missed a non-trivial portion of patients, who later developed cancer. All microscopic slides were independently evaluated by two pathologists, and in the rare cases of discrepant initial scores, a final score was agreed on after discussion.

### Statistics

The pathology scores assess staining intensity and percent positivity. The predictors were each categorical (pathology scores 0–3) or dichotomized (pathology scores low versus high). Statistical analysis was done with the t test and calculation of odds ratios with confidence intervals.

## Results

### Patient characteristics

The papillary breast lesions encompass a spectrum of masses, which present as fronds attached to the inner mammary duct wall by a fibrovascular core with both epithelial and myoepithelial cells; although not malignant, papillary disease is associated with a risk of progression to invasive breast cancer [1, 24, 25]. From the patients seen at the two locations involved, 114 paraffin blocks and follow-up information were available (Table 1). These women grouped into those who did not experience cancer over at least 5 years, those who progressed to cancerous lesions in the same breast (12 patients or 10.5%, slightly higher than most literature estimates for progression risk), and those who later encountered cancer in the contralateral breast (6 patients or 5.3%).

**Table 1:** Patient characteristics

	*n*	Cincinnati	Wroclaw	Age (Cin)	Age (Wroc) mean ± std	Progression years (range)	Follow-up years (range)
Progression	12	4	8	Unknown	63.6 ± 13.1	0.83–11	–
Contralateral cancer	6	6	0	Unknown	–	4.92–10.92	–
No malignancy of the breast	96	55	41	Unknown	58.0 ± 12.4	–	5.0–10.9

Archival breast specimens were obtained from the Universities of Cincinnati and Wroclaw. All patients belonged to three groups, those who remained cancer free, those whose lesions progressed to breast cancer, and (from the Cincinnati contingent) those who later were diagnosed with cancer in the contralateral breast. Age information was available from the Wroclaw patients. *n* = total number of samples, std = standard deviation. The follow-up for patients, who developed cancer, was until the time of diagnosis

### Immunohistochemistry

**Fig. 2:**
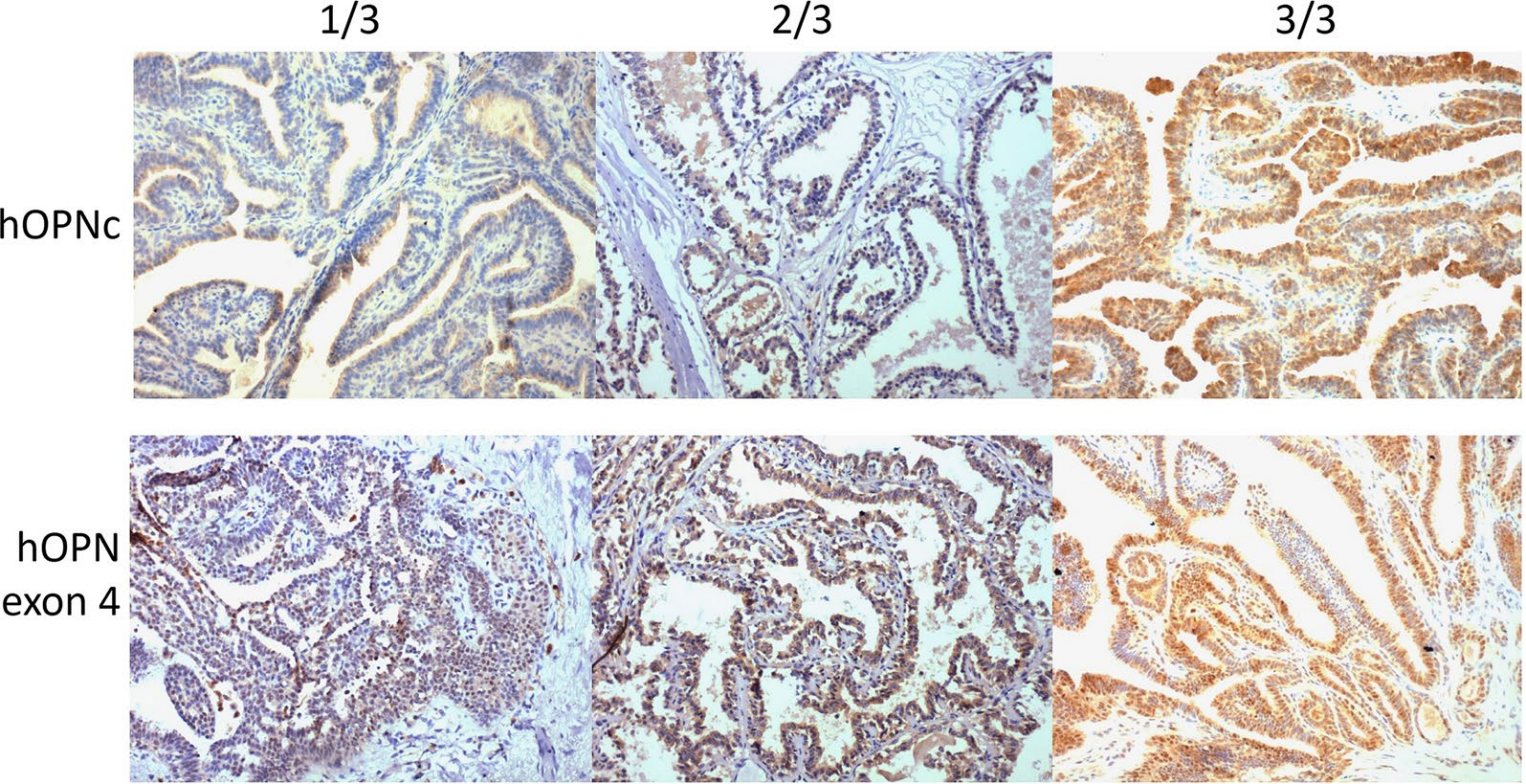
Immunohistochemistry. Representative staining for OPN-c and OPN-exon-4, counterstained with hematoxylin, original magnification 100x. The pathology scores for intensity/percent positivity are indicated above the slides. (Top panel) hOPNc stained with antibody clone F2. (Bottom panel) hOPN-exon-4 stained with MAB193P

All lesions had two sections prepared for staining with monoclonal antibodies to either OPN-c or OPN-exon-4 (Fig. 2). The signal for Osteopontin-c was cytoplasmic, whereas Osteopontin exon 4 stained both cytoplasm and nucleus. In each case, only the papillomatous lesion was scored for intensity and percent positivity.

### Prognosis

**Fig. 3:**
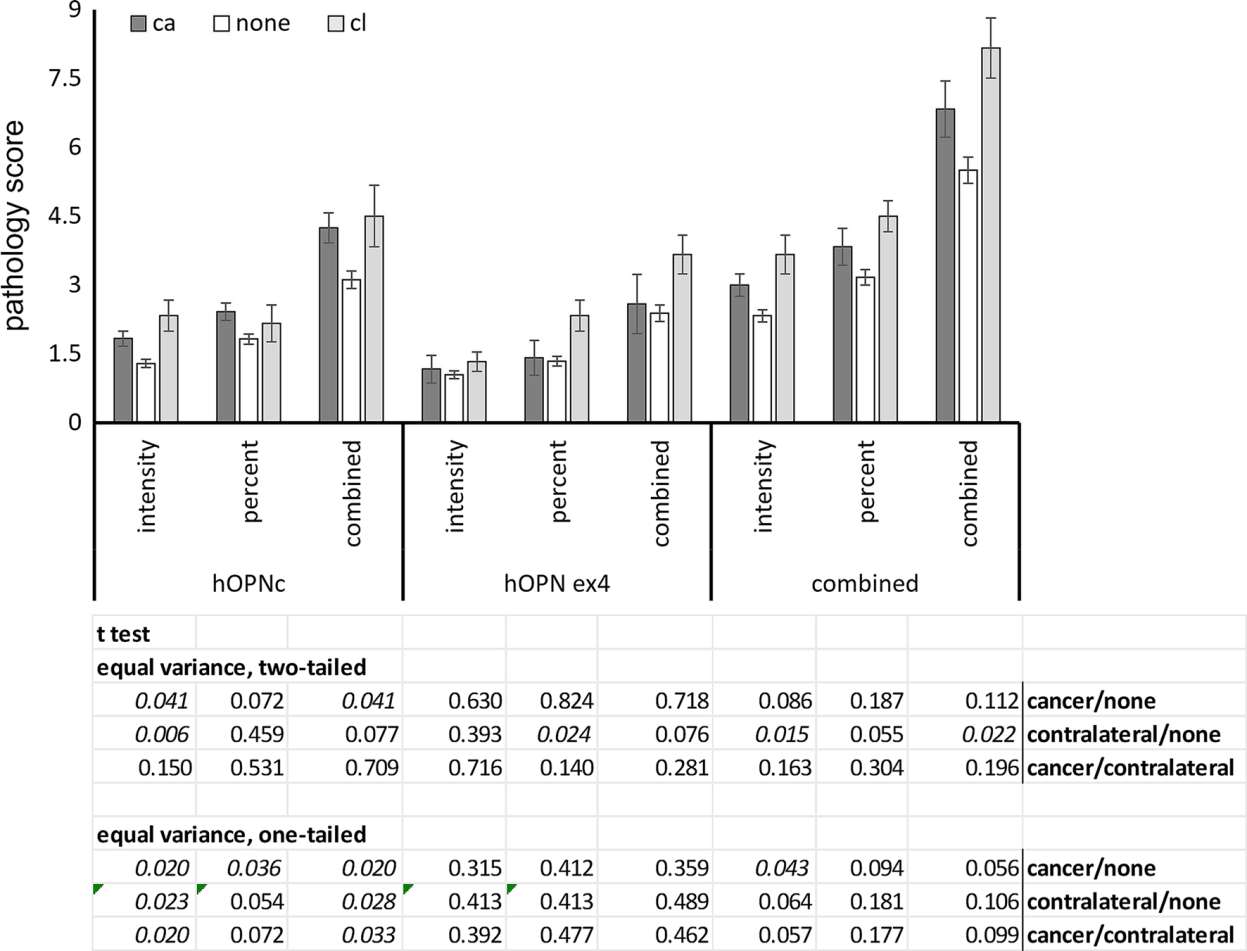
Osteopontin splice variants are indicators for prognosis. The bar graph shows mean ± standard error of the pathology scores (staining intensity, percent positivity, additive score) for OPN-c (antibody F2), OPN-exon-4 (antibody MAB193P), evaluation of both combined together. The additive scores were obtained by adding their component scores (intensity plus percent positivity). ca = cancer developed, none = no progression, cl = later diagnosis of cancer in the contralateral breast. The bottom panel shows the p-values for two-tailed and one-tailed t tests (assuming elevation in the progressors) for comparisons among the patient groups. Values below 0.05 are displayed in italics

The pathology scores covered a range, with none of the patients who later experienced cancer in either breast having intensity or percent positivity of 0. The OPN-c intensity scores were significantly higher in women who experienced cancer at a later time than in women who remained free of progression. This was the case for a two-tailed t test (assuming equal variance) (*p* = 0.041) and for a one-tailed *t* test assuming that in progression the direction of change is an increase in the pathology score (*p* = 0.020). The exon 4 staining contributed very little, with the staining intensity not differing significantly among the patient groups. Only the percent positivity was marginally significantly elevated (*p* = 0.024, two-tailed t test, but not significant in the one-tailed t test) for patients with later cancer in the other breast (Fig. 3).

**Fig. 4:**
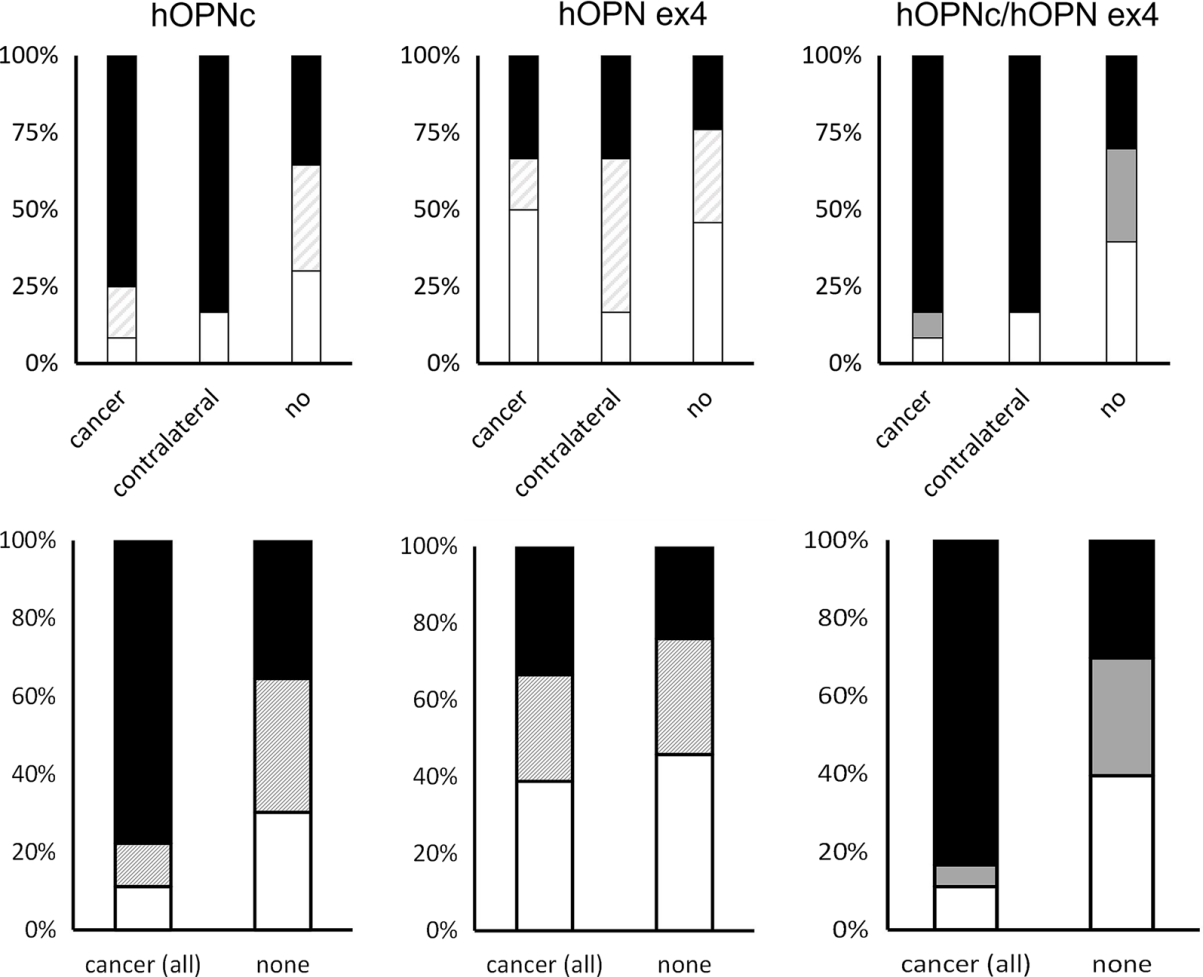
Breast cancer risk score. For the analysis of OPN-c (left) and OPN-exon-4 (middle), the pathology scores were condensed as 0,1 = low and 2,3 = high. Then, an additive score was derived as intensity plus percent positivity with low (open) = both contributors low, intermediate (hatched) = one low and the other high, high (filled) = both high (as marked in the left graph on the top). To evaluate both markers in conjunction, a combined score (right) was derived from the additive scores for both antibodies, such that low/low and low/intermediate were combined to low (open), low/high and intermediate/intermediate were combined to intermediate (gray), and intermediate/high and high/high were combined to high (filled) (as marked in the top right graph). Top panel) The groups comprise those who remained cancer free, those whose lesions progressed to breast cancer, and those who later were diagnosed with cancer in the contralateral breast. hi = high pathology score, int = intermediate pathology score, lo = low pathology score. Bottom panel) Cancer risk combined for ipsilateral or contralateral breast versus no progression

Previously [17], we found it helpful to develop a risk score, in which we added the pathology scores for intensity and percent positivity followed by ranking into low, intermediate, or high for each marker. We could then generate a combined score for several markers (specifically OPN-c and OPN-exon-4) using the analogous process of addition followed by categorization. About 75% of women who later developed cancer in the same breast had high OPN-c staining at the time of papilloma biopsy; for later cancer in the other breast this percentage was over 80. OPN-exon-4 immunohistochemistry was only marginally discriminating for cancer in the same breast, but may have a substantially reduced fraction of low-risk scores when cancer is later experienced in the contralateral breast (Fig. 4, for various cutoffs, see Additional file 1: Figure S1). For low, intermediate, or high additive OPN-c pathology scores, the cancer risk increased 3.2%, 5.7%, 18.8% for the same breast and 3%, 0%, 10% for the contralateral breast. A risk progression was less clear for the staining with anti-OPN-exon-4 antibody or when merging the two markers into a combined risk score (Table 2). The relative risk for progression in the ipsilateral (RR 4.043, CI 95% 1.159–14.109) or contralateral (RR 7.143, CI 95% 0.866–58.946) breast was significantly elevated in the high versus low comparison for OPN-c intensity (Table 3; the relative risk by pathology score is shown in Additional file 1: Table S1). The overall incidence of progression for papilloma is about 10.5% (and for cancer in the other breast about 5.3%).

**Table 2:** Prognosis for cancer by breast

	hOPNc additive		hOPNex4 additive			both combined	
	% progr	*n*	% progr	*n*		% progr	*n*
A							
Low	3.2	31	11.8	51	L/L, L/I	0.0	39
Intermediate	5.7	35	5.9	34	L/H, I/I	21.1	38
High	18.8	48	13.8	29	I/H, H/H	10.8	37
All	10.5	114	10.5	114	All	10.5	114
B							
Low	3	31	2	51	L/L, L/I	3	39
Intermediate	0	35	9	34	L/H, I/I	3	38
High	10	48	7	29	I/H, H/H	11	37
All	5	114	5	114	All	5	114

The pathology scores were condensed to 0,1 = low and 2,3 = high. Then, an additive score was derived as intensity plus percent positivity with low = both components low, intermediate = one low and the other high, high = both high. hOPNc additive = additive score for staining with anti-OPNc antibody F2, hOPNex4 additive = additive score for staining with anti-exon 4 antibody MAP193P. A combined score was derived from the additive scores for both antibodies, such that low/low and low/intermediate were combined to low, low/high and intermediate/intermediate were combined to intermediate, and intermediate/high and high/high are combined to high. % progr. Indicates the proportion of patients (in percent), who experienced cancer; the adjacent column (*n*) indicates the number of patients in the group. The bottom row is reflective of the total number of patients under study. **A** Progression in the ipsilateral breast. **B** Later cancer in the contralateral breast

**Table 3:** Relative risk for progression to cancer

Ipsilateral							Contralateral						
	Comparison	Relative Risk	CI (95%)		*p* value Fisher	*p* value *χ*^2^		Comparison	Relative risk	CI (95%)		*p* value Fisher	*p* value *χ*^2^
**OPN-c**							**OPN-c**						
Intensity	H/L	4.043	1.159	14.109	0.027	0.016	Intensity	H/L	7.143	0.866	58.946	0.079	0.031
Positivity	H/L	4.484	0.651	35.970	0.101	0.008	Positivity	H/L	1.000	0.193	5.190	1.000	1.000
Additive	(I + H)/L	4.231	0.571	31.366	0.173	0.111	Additive	(I + H)/L	2.083	0.254	17.087	0.668	0.480
	H/I	3.663	0.846	15.863	0.099	0.055		H/I	(0.128)			0.058	0.033
**OPN ex4**							**OPN ex4**						
Intensity	H/L	1.623	0.556	4.743	0.507	0.375	Intensity	H/L	1.200	0.232	6.205	1.000	0.828
Positivity	H/L	0.769	0.260	2.274	0.763	0.634	Positivity	H/L	4.808	0.582	39.703	0.205	0.102
Additive	(I + H)/L	0.862	0.297	2.504	1.000	0.785	Additive	(I + H)/L	3.947	0.478	32.597	0.225	0.163
	H/I	2.296	0.456	11.569	0.402	0.297		H/I	0.853	0.154	4.228	1.000	0.855
**Combined**							**Combined**						
Intensity	(I + H)/L	(0.182)			0.003	0.003	Intensity	(I + H)/L	3.644	0.441	30.080	0.397	0.192
	H/I	0.680	0.167	2.771	0.719	0.580		H/I	1.956	0.036	10.603	0.593	0.434
Positivity	(I + H)/L	(0.136)			0.118	0.580	Positivity	(I + H)/L	(0.073)			0.595	0.434
	H/I	0.689	0.224	2.119	0.754	0.511		H/I	1.278	0.274	5.959	1.000	0.755

The relative risk for progression was calculated for the ipsilateral (left block) or contralateral (right block) breast. The pathology scores were condensed to 0,1 = low (L) and 2,3 = high (H). Then, an additive score was derived as intensity plus percent positivity with low = both components low, intermediate (*I*) = one low and the other high, high = both high. hOPNc additive = additive score for staining with anti-OPNc antibody F2, hOPNex4 additive = additive score for staining with anti-exon 4 antibody MAP193P. A combined score was derived from the additive scores for both antibodies, such that low/low and low/intermediate were combined to low, low/high and intermediate/intermediate were combined to intermediate, and intermediate/high and high/high are combined to high. CI (95%) = 95% confidence interval, Fisher indicates Fisher’s exact test, *c*^2^ = *c*^2^ test. Numbers in parentheses indicate the risk for the higher pathology score alone (relative risk could not be calculated because of a division by 0 error)

In multi-year follow-up, patients with mammary papilloma have sometimes displayed cancerous lesions in the contralateral breast [10]. In this study, highly staining lesions were predictors for contralateral breast malignancy (Fig. 4, Table 2), although over a longer follow-up (8 ± 2 years, mean ± range). This may suggest that there are genetic factors, which predispose to elevated Osteopontin splice variant expression. The resulting abundance of Osteopontin variants increases the progression risk, when early transformed lesions occur in either breast.

## Discussion

A recent review states: “When cancer is detected at the earliest stages, treatment is more effective and survival drastically improves. Yet ~ 50% of cancers are still only detected at an advanced stage. (…) [One] challenge is to build a greater understanding of the biology and behavior of early disease. This will help identify ways to distinguish between consequential, aggressive lesions and inconsequential lesions that will not cause harm” [26]. The presence of Osteopontin splice variants can differentiate early lesions in this manner. In the present study, we have identified the Osteopontin splice variant-c as a prognostic indicator for ensuing invasive disease following papillomatous growths in the breast. Distinguishing high-risk patients from low-risk patients will improve the prognosis of the former group (through early decisive intervention) and spare unnecessary treatment for the latter group (through watchful waiting).

The association of Osteopontin with cancer progression has been reported in the literature for decades. However, the complexity of the molecule has precluded its development as a diagnostic. The protein is subject to phosphorylation and glycosylation, calcium, and heparin binding, as well as cleavage by various proteases. In ELISA setups, this malleability has previously caused inconsistencies in the readouts [27]. The focus on alternatively spliced forms of the cytokine has multiple advantages, including the lack of posttranslational modifications in proximity to the splice sites and the absence of splice variants from untransformed tissues.

Seemingly discordant with prior findings in other premalignant breast lesions [17], OPN-exon-4 did not substantively contribute to the assessment of progression risk in the same breast. This raised the question whether the lack of exon 4 contribution to the risk score is a characteristic of the papillomatous lesions, reflects a difference between the previously used polyclonal antibodies and the monoclonals applied here, is an indication of limited power (as only 114 papilloma cases were available for investigation), or has other explanations. The predecessor analysis of 434 cases (covering no lesion, usual ductal hyperplasia, radial scar, atypical ductal hyperplasia, papillomatosis, lobular carcinoma in situ, ductal carcinoma in situ) included 69 progressions by other lesions, but had no progression among the 6 papillomas covered in the study. In that report, the inclusion of exon 4 considerably improved the prognostication of future deaths but contributed only marginally to the prognostication of progression to cancer (which could mostly be based on Osteopontin-c). Whereas the expression of Osteopontin in mammary tissue is estrogen-inducible, splicing of its RNA message is absent from healthy breasts. The data from the available studies suggest that the form OPN-c is the dominant indicator for progression by premalignant lesions. While it is itself sufficient to assess progression risk, there may be benefit in combining OPN-c with other markers that can provide additional information, such as estrogen receptor [28], HER-2 [29], or OPN-exon-4.

The Cincinnati patients included 6 individuals, who later developed breast cancer in the other breast. Their Osteopontin-c levels were substantially elevated compared to later cancer-free patients (as shown in Fig. 3). For these women, the contribution from the additional exon 4 staining (mostly in terms of percent positivity) seemed to contribute more markedly to the prognosis assessment than was seen for the ipsilateral recurrences. It is reasonable to hypothesize that elevated expression of Osteopontin and its variants in papillomas may be subject to genetic predisposition, such as promoter polymorphisms [30] or interindividual heterogeneity in the splicing machinery, which impose an elevated risk on lesions that might otherwise remain benign and undetected. Therefore, women, who encounter one premalignant lesion with high Osteopontin-c and Osteopontin exon 4, will likely express these molecules in future breast lesions (located in either breast), thus rendering those growths high-risk for full transformation to breast cancer.

Some open questions remain to be addressed and will benefit from a larger, multi-center follow-up (the low incidence of breast papilloma puts strains on sample availability). While the monoclonal antibodies had been raised to the same epitopes as their polyclonal predecessors (the antibody to splice variant-c recognizes a 9-mer amino acid sequence), the subcellular preference of the staining changed. The monoclonal signal for OPN-c is clearly cytosolic, while the monoclonal exon 4 signal covers cytoplasm and nucleus. Regardless, here we show that the anti-OPN-c antibody has preserved its prognostic characteristics.

## Supplementary Information

**Additional file 1**. **Table S1:** Relative risk based on pathology scores. **Figure S1:** Breast cancer risk scores.

## Data Availability

Data are available upon request.
