# Supplementary material for: Breast cancer risk in papilloma patients: Osteopontin splice variants indicate prognosis
Source: Breast Cancer Res. 2022 Sep 29;24:64. doi: 10.1186/s13058-022-01561-9 (PMC9520814; doi:10.1186/s13058-022-01561-9)
Supplement: Supplementary file 1 — Additional file 1. Table S1: Relative risk based on pathology scores. Figure S1: Breast cancer risk scores. [file 13058_2022_1561_MOESM1_ESM.pdf]

Table S1: Relative risk based on pathology scores. The relative risk for progression was calculated for the ipsilateral (upper block) or contralateral (lower block) breast. + behind a score indicates all scores equal to and higher than the given number lumped together. CI(95%) = 95% confidence interval, Fisher indicates Fisher's exact test, χ^2 = χ^2-test. Numbers in parentheses indicate the risk for the higher pathology score alone (relative risk could not be calculated because of a division by 0 error).

ipsilateral

OPNc

	comparison	Relative Risk	CI (95%)		p-value Fisher	p-value χ^2
intensity	1+ vs 0	(0.133)			0.211	0.100
	2+ vs 1	2.870	0.831	9.912	0.120	0.075
	3 vs 2	0.450	0.064	3.186	0.659	0.389
positivity	1+ vs 0	(0.133)			0.211	0.100
	2+ vs 1	2.200	0.307	15.785	0.682	0.405
	3 vs 2	1.108	0.370	3.318	1.000	0.855
additive	1+ vs 0	(0.133)			0.211	0.100
	2+ vs 1					
	3+ vs 2	1.692	0.237	11.952	1.000	0.584
	4+ vs 3	2.000	0.467	8.567	0.487	0.329
	5+ vs 4	1.724	0.480	6.192	0.480	0.393
	6 vs 5	0.444	0.060	3.277	0.633	0.393

OPN ex4

	comparison	Relative Risk	CI (95%)		p-value Fisher	p-value χ^2
intensity	1+ vs 0	0.805	0.261	2.481	0.740	0.707
	2+ vs 1	2.222	0.571	8.644	0.277	0.236
	3 vs 2	1.813	0.264	12.436	0.500	0.558
positivity	1+ vs 0	0.805	0.261	2.481	0.740	0.707
	2+ vs 1	0.801	0.208	3.089	0.710	0.748
	3 vs 2	8.235	0.995	68.152	0.034	0.018
additive	1+ vs 0	0.081	0.261	2.481	0.740	0.707
	2+ vs 1					
	3+ vs 2	0.983	0.216	4.467	1.000	0.982
	4+ vs 3	3.286	0.410	26.342	0.386	0.224
	5+ vs 4	3.273	0.634	16.883	0.297	0.137
	6 vs 5	2.250	0.355	14.279	0.491	0.425

combined

	comparison	Relative Risk	CI (95%)		p-value Fisher	p-value χ^2
intensity	0 vs 1+	(0.118)			1.000	0.373
	1 vs 2+	(0.146)			0.117	0.069
	2 vs 3+	1.417	0.464	4.329	0.753	0.536
	3 vs 4+	1.087	0.307	3.851	1.000	0.897
	4 vs 5+	0.000			1.000	0.394
	5 vs 6					
positivity	0 vs 1+	(0.118)			1.000	0.373
	1 vs 2+	(0.130)			0.602	0.224
	2 vs 3+	1.765	0.416	7.486	0.725	0.425
	3 vs 4+	0.500	0.160	1.563	0.254	0.236
	4 vs 5+	1.517	0.341	7.548	0.688	0.606
	5 vs 6	2.625	0.441	15.609	0.300	0.280

contralateral

OPNc

	comparison	Relative Risk	CI (95%)		p-value Fisher	p-value χ^2
intensity	1+ vs 0	(0.071)			0.587	0.242
	2+ vs 1	5.000	0.610	40.995	0.202	0.090
	3 vs 2	3.750	0.714	19.707	0.131	0.097
positivity	1+ vs 0	(0.071)			0.587	0.242
	2+ vs 1	0.471	0.094	2.349	0.320	0.355
	3 vs 2	2.667	0.292	24.370	0.616	0.362
additive	1+ vs 0	(0.071)			0.587	0.242
	2+ vs 1					
	3+ vs 2	0.833	0.106	6.527	1.000	0.863
	4+ vs 3	1.878	0.222	15.872	1.000	0.553
	5+ vs 4	(0.148)			0.117	0.060
	6 vs 5	1.700	0.282	10.260	0.613	0.561

OPN ex4

	comparison	Relative Risk	CI (95%)		p-value Fisher	p-value χ^2
intensity	1+ vs 0	(0.08)			0.337	0.130
	2+ vs 1	0.750	0.146	3.841	1.000	0.728
	3 vs 2	0.000	0.000	0.000	1.000	0.626
positivity	1+ vs 0	(0.080)			0.740	0.707
	2+ vs 1	2.212	0.273	17.883	0.660	0.438
	3 vs 2	3.375	0.623	18.282	0.163	0.136
additive	1+ vs 0	(0.08)			0.337	0.130
	2+ vs 1					
	3+ vs 2	1.579	0.197	12.646	1.000	0.661
	4+ vs 3	2.706	0.323	22.689	0.638	0.331
	5+ vs 4	0.926	0.110	7.800	1.000	0.943
	6 vs 5	0.000	0.000	0.000	1.000	0.708

combined

	comparison	Relative Risk	CI (95%)		p-value Fisher	p-value χ^2
intensity	0 vs 1+	(0.063)			1.000	0.528
	1 vs 2+	(0.079)			0.338	0.194
	2 vs 3+	3.444	0.423	28.064	0.391	0.210
	3 vs 4+	3.826	0.463	31.619	0.346	0.170
	4 vs 5+	1.583	0.216	11.581	1.000	0.659
	5 vs 6	0.000	0.000	0.000	1.000	0.248
positivity	0 vs 1+	(0.063)			1.000	0.528
	1 vs 2+	(0.070)			1.000	0.388
	2 vs 3+	(0.094)			0.331	0.136
	3 vs 4+	(0.118)			0.333	0.194
	4 vs 5+	0.444	0.089	2.214	0.402	0.306
	5 vs 6	2.857	0.205	39.829	0.459	0.419

Figure S1: Breast cancer risk scores. Comparison among three different cut-offs for hOPN-c intensity scores to dichotomize low versus high. The left panel shows the chosen separation of 0,1 = low and 2,3 = high, the middle panel displays 0 = low and 1-3 = high, the right panel has 0-2 = low and 3 = high.

Figure S1

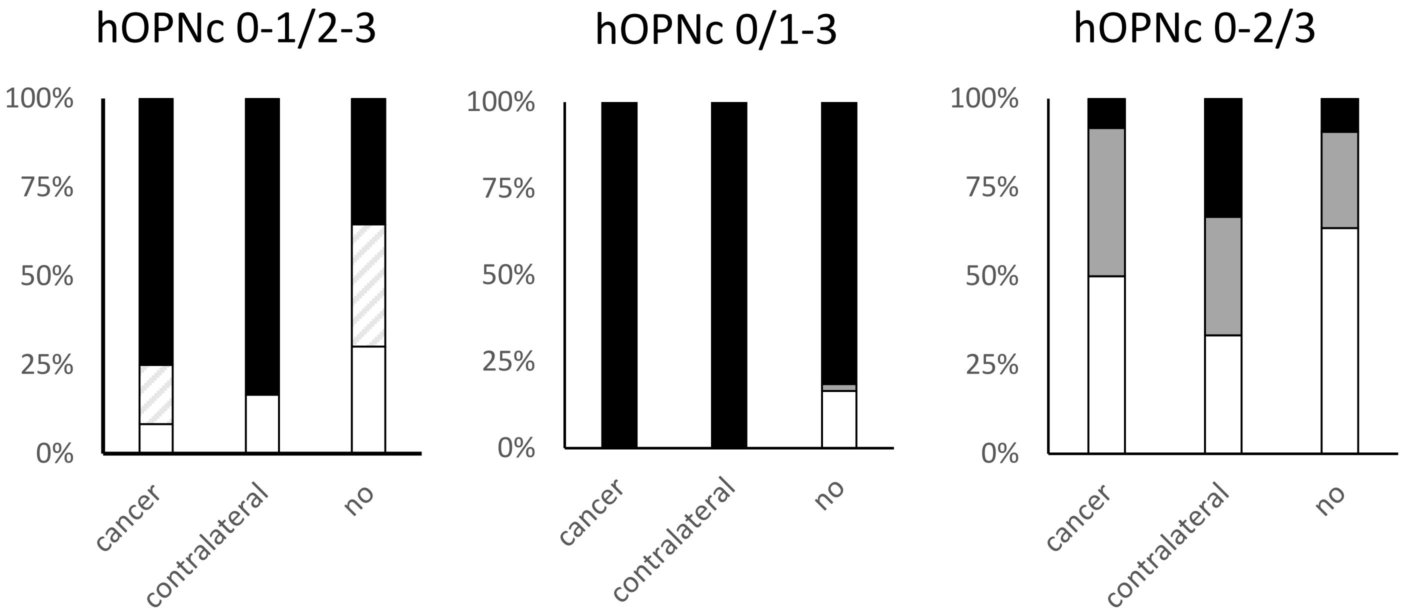
hOPNc 0-1/2-3
100%
75%
50%
25%
0%
cancer
contralateral
no
hOPNc 0/1-3
100%
75%
50%
25%
0%
cancer
contralateral
no
hOPNc 0-2/3
100%
75%
50%
25%
0%
cancer
contralateral
no
